# Infrastructure and Educational Needs of Newborn Screening Short-Term Follow-Up Programs within the Southeast Regional Newborn Screening & Genetics Collaborative: A Pilot Survey

**DOI:** 10.3390/healthcare3040964

**Published:** 2015-10-15

**Authors:** Cecelia A. Bellcross, Lokie Harmond, Phaidra Floyd-Browning, Rani Singh

**Affiliations:** 1 Emory University School of Medicine, Atlanta, GA 30322, USA; E-Mails: lokieh@yahoo.com (L.H.); rsingh@emory.edu (R.S.); 2 Tulane University, New Orleans, LA 70118, USA; E-Mail: pfloyd@samford.edu

**Keywords:** newborn screening, short-term follow-up, long-term follow-up, protocols, education

## Abstract

Newborn screening (NBS) follow-up protocols vary significantly by state, and there is a need to better understand the infrastructure and communication flow of NBS programs. In addition, assessment of the educational needs of families and providers with regard to the implications of NBS results is required to inform the development of appropriate informational resources and training opportunities. To begin to address these issues, we administered a web-based survey to state NBS coordinators within the Southeast Regional Newborn Screening & Genetics Collaborative (SERC). Fourteen coordinators responded to the survey, including at least one from each of the 10 SERC states/territories. Over one-third of respondents had never received formal training regarding the metabolic conditions identified on NBS. Most communicated results via telephone or fax, though two centers indicated use of a web-based platform. Only two programs were involved in directly reporting results to the family. Four programs reported a long-term follow-up protocol. Deficits were noted for primary care provider (PCP) knowledge of metabolic disorders identified on NBS, and how to inform parents of abnormal results. Close to half indicated that the adequacy of the number of genetic counselors, dietitians, and medical/biochemical geneticists was minimal to insufficient. Respondents uniformly recognized the importance of providing additional educational and informational resources in multiple categories to NBS staff, PCPs, and families.

## 1. Introduction

Newborn screening (NBS) was launched by the United States (U.S.) as a public health initiative in the in the 1960s with Phenylketonuria (PKU) as the first condition [1]. NBS has expanded since then to many countries, and many more disorders. While primarily focused on inborn errors of metabolism, NBS also includes non-metabolic conditions such as hearing loss and congenital heart disease. The premise of NBS is to identify these conditions in newborn infants when early treatment and intervention can prevent morbidity and mortality and improve long-term outcomes.

With the increasing number of conditions identifiable on newborn screening, the U.S. Centers for Disease Control and Prevention (CDC) and the Health Resources and Services Administration (HRSA) have recognized the need for high-quality research and clinical expertise to maximize patient care and outcomes [2]. To achieve these goals, seven regional genetics and newborn screening collaboratives were formed in the U.S. under the support of HRSA [3]. The Southeast Regional Newborn Screening & Genetics Collaborative (SERC, Region 3) includes the states of Alabama, Florida, Georgia, Louisiana, Mississippi, North Carolina, South Carolina, Tennessee, and the territories of Puerto Rico, Virgin Islands. The primary aims of SERC are to promote translation of healthcare research into practice and to improve care for individuals with heritable disorders in the southeast region of the U.S. [4].

Each individual state/territory has its own legislative mandate regarding NBS, which defines both the conditions screened for and policies regarding reporting and follow-up. No national guidelines exist for these processes. While most states screen for a common panel of 29 metabolic conditions [5], limited data exist regarding how NBS results for metabolic conditions are relayed to primary care providers, variation in short-term protocols between states, and the educational needs of both those relaying and receiving the information [6]. A survey of state reporting mechanisms, focused on ability to accommodate emergency situations, demonstrated variability of methods used to deliver NBS test results to the responsible entities [7]. The issue of protocols and uniformity was also recently evaluated in the European Union [8]. Data from 37 countries revealed variability in screening panels, as well as limited and inconsistent materials to inform parents about the results and necessity of confirmatory diagnosis and treatment. A minority of countries offered training for pediatricians (40%), and dietitians (29%), and rarely for other professions. Only 3% conducted registry-based evaluation of long-term outcomes [8].

We hypothesize that similar variability of follow-up protocols and deficiencies of training and educational resources exist in the SERC and other regions. The purpose of this study is to initially assess NBS follow-up protocols, methods of communication, and need for education/training in the SERC states/territories. Results from evaluating these issues in the SERC will guide development of a nation-wide survey, as well as provide a basis for designing educational materials and methods of delivery to improve patient care.

## 2. Methods

Publically available information was used to contact the NBS coordinating center of all 10 states/territories in SERC and verify the names and email addresses of individuals involved with NBS short-term follow-up for administration of a web-based survey. The survey was designed to assess the existing infrastructure, protocols, and educational needs of NBS programs within the SERC. Although we asked if the programs had a formal long-term follow-up protocol, the survey focused primarily on the short-term follow-up of inherited metabolic conditions identified by analysis of newborn dried blood spots (NDBS). In this context, short-term follow-up refers to the process of ensuring that all newborns are screened, that an appropriate follow-up caregiver is informed of results, that confirmatory testing has been completed, and that the infant has received a diagnosis and, if necessary, treatment.

The survey was administered electronically via Survey Monkey, and included a total of 35 questions divided into the following sections:

- Coordinator Information: Profession/background, years involved in NBS follow-up and level of formal training (courses, conferences, targeted workshops) received regarding inherited metabolic diseases identified on NBS.
- Program Information: Metabolic conditions screened for, professionals involved directly with NBS in the state, short-term follow-up protocols, conduction of long-term follow-up.
- NBS Resources and Educational Needs: Perceived adequacy of the number of healthcare professionals directly involved in NBS follow-up, perceived adequacy of the knowledge base/expertise of these professionals as well as primary care providers (PCPs). The type of information provided to PCPs and families, perceived educational and resource needs of NBS staff, PCPs, and families, and preferred methods of delivery.

Questions regarding perceived adequacy and educational/resources needs were measured on a five-point Likert scale. Descriptive statistics were evaluated. This study was approved by the Institutional Review Board of Emory University.

## 3. Results

A total of 14 individuals out of the 17 contacted (82%) completed all/most of the survey, including at least one individual from each of the 10 states/territories. Respondents included six nurses, seven public health professionals, and one genetic counselor. Regarding professional experience with NBS screening, four respondents reported 1–5 years, five reported 6–10 years, two reported 11–19 years, and three reported 20 years or more. Only five of the 14 had ever received formal training (courses, conferences, targeted workshops) regarding the metabolic conditions identified on newborn screening. Among the 10 states/territories, four indicated they had some formal long-term follow-up process. All states/territories except one conducted the short-term follow-up in-house *vs.* contracting out. Four of the 10 states/territories do not screen for all conditions on the HRSA recommended Uniform Screening Panel Core Conditions [5]. With respect to reporting abnormal NBS results, eight used non-automated phone calls or fax with just two indicating use of a web-based reporting system. All 10 states/territories notify the PCP (assumed in most cases to be pediatricians or family practitioners), and eight indicated the PCP is primarily responsible for informing the family, while two indicated NBS program staff notifies the family directly of abnormal NBS results. Seven of the 10 states/territories reported use of a reminder system to monitor whether appropriate follow-up is being completed. Referral services and resources provided to parents included genetics/metabolic clinic (8/10), nutrition/dietitian and Medicaid/WIC (6/10), Birth-to-Three programs (5/10), disease related materials and support group information (3/10). A minority of programs (4/10), send written information directly to parents regarding their child’s specific disease, false positive results, or carrier implications. Resources used in diagnostic follow-up of abnormal NBS results included the American College of Medical Genetics (ACMG) Act Sheets (8/10) and diagnostic algorithms (3/10) [9]. Four states reported use of Screening, Technology and Research in Genetics (STAR-G) resources [10] and three states reported using materials developed in-house.

We queried respondents on their perceived adequacy of the number of professionals in their state involved in NBS follow-up and care. Minimal to insufficient was indicated by 4/11 for NBS staff, 5/12 for dietitians/nutritionists, 5/8 for genetic counselors, 3/8 for medical geneticists, and 3/8 for clinical biochemical geneticists. However the majority (mean 74%) of respondents indicated that the knowledge base of these professionals to conduct appropriate NBS follow-up was very good to good.

**Figure 1:**
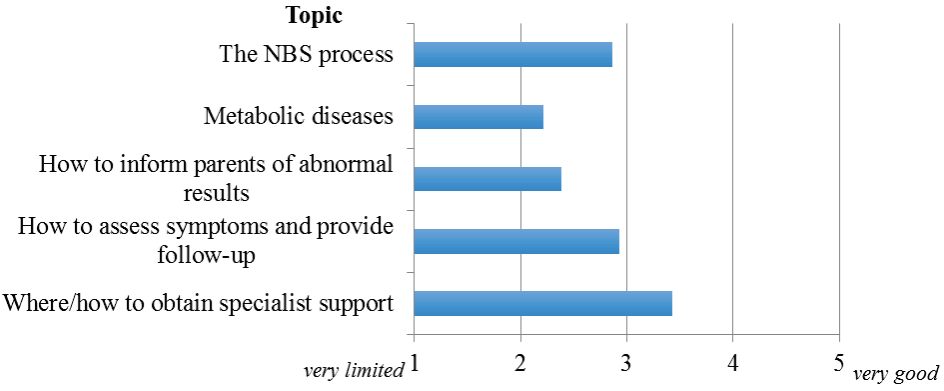
Perceived knowledge-base of PCPs.

Respondents were asked to indicate their perception of PCP knowledge regarding the following NBS topics: the NBS process, metabolic disorders identified on NBS, how to inform parents of abnormal screening results, how to assess patient symptoms and provide appropriate follow-up, and where/how to obtain specialist support. They were asked to provide their impressions of the “average” PCP in their state/territory relative to the needs of their protocol and the patients/families ranging from very limited to very good. Figure 1 illustrates participant responses regarding PCP knowledge in these areas. The two where PCP knowledge was considered most deficient were metabolic diseases identified on NBS (mean rating 2.2) and how to inform parents of results and implications (mean rating 2.4). The only area where PCP knowledge was judged as ‘good’ to ‘very good’ by the majority of respondents (mean rating 3.5) was where/how to obtain specialist support. Interestingly, 7/10 states/territories indicated they routinely provide PCPs with information and resources regarding all or some of these topics. The most common method of dissemination of this information is via mailed or faxed written materials (81%) though 72% also indicated utilization of websites.

**Figure 2:**
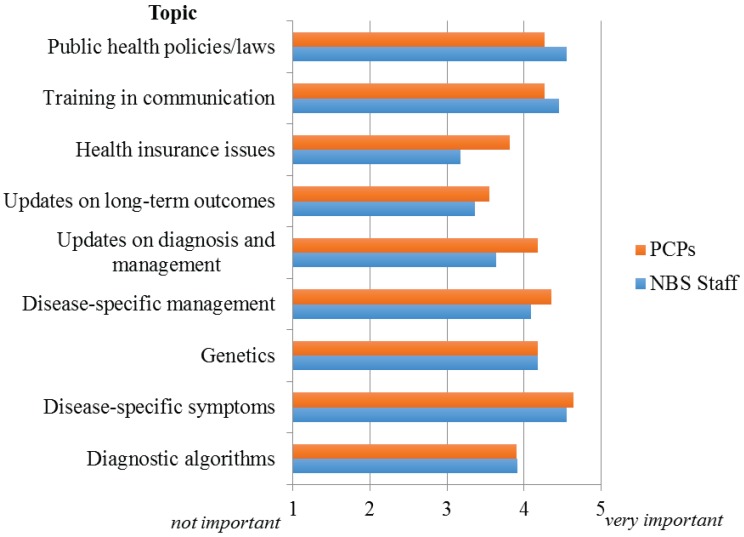
Perceived importance of providing educational and information resources to NBS staff and PCPs.

Participants were also asked to rank their perception of the relative importance of providing educational and informational resources specifically regarding the metabolic conditions identified on NBS to both NBS staff and PCPs. Figure 2 illustrates the responses to this question for the topics queried. For both NBS staff and PCPs, the most important area identified was disease-specific symptoms and manifestations. For dissemination of this information to NBS staff, webinars and educational conferences were selected as the preferred methods of delivery; while for PCPs it was grand rounds/seminars.

**Figure 3:**
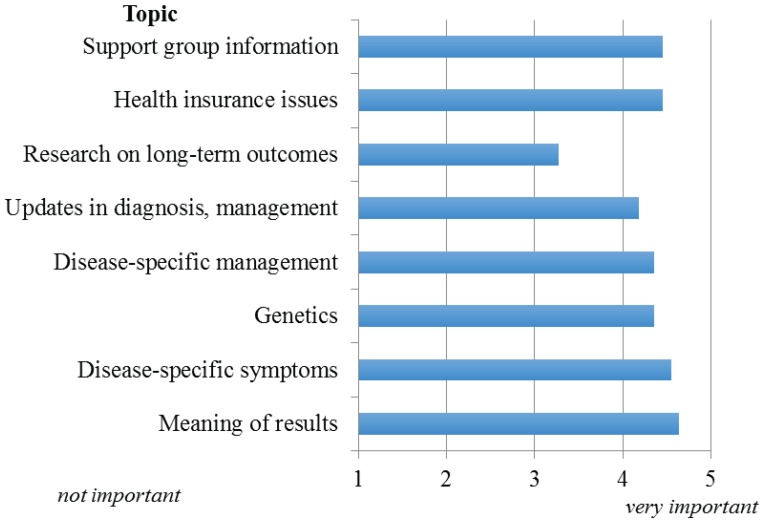
Importance of information to be provided to parents/families of infants with abnormal NBS result.

The relative importance of information to provide parents/families of infants with abnormal NBS results is given in Figure 3. With the exception of research regarding long-term follow-up and outcomes, all topics were considered either very important or important to relay, with information regarding the meaning of results (positive, negative, carrier) considered the most important. Respondents indicated electronic media (apps, YouTube, text services) followed by websites would be the preferred means of providing families access to this information.

## 4. Discussion and Conclusions

The increasing number and complexity of the metabolic conditions identified on NBS creates challenges for NBS staff and PCPs involved in the care of these infants, as well as the parents faced with understanding the meaning and implications of their child’s diagnosis. We surveyed those involved on the front line of disseminating NBS results in the SERC—the short-term follow-up coordinators—about their follow-up protocols; as well as perceived knowledge base of NBS staff and PCPs regarding the metabolic conditions identified on NBS, and educational needs of NBS staff, PCPs and parents/families. Similar to previous studies in the U.S. [7] and European Union [8] we found variability of NBS protocols among the 10 states/territories that comprise the SERC, limited training of NBS staff and PCPs, and inconsistent provision of resources to PCPs and families. The finding that 40% conduct some form of long-term follow-up is higher than that reported in Europe, though details on these protocols were not obtained [8]. For many of the newer conditions added to NBS, data on long-term outcomes will be essential for determining the efficacy of screening and providing prognostic information for families. As noted by the U.S. Secretary for Health and Human Services’ Advisory Committee, systematic long-term follow-up of health outcomes for disorders identified on NBS is in the very early stages of development [11].

Despite the increasing advent of web-based tools to relay information, the majority of programs are still using phone calls or fax to disseminate NBS results. A nationwide survey conducted in 2009 found approximately 40% of programs used web/online methods of reporting, and stressed the need for uniform web-based communication, specifically with respect to emergency preparedness [7]. There is also a concerning perception among NBS personnel of deficiencies in PCP knowledge regarding the inherited metabolic conditions screened for, as well as how to assess symptoms and provide follow-up. Our findings also suggest training is needed for PCPs regarding how to best inform families of abnormal NBS results, as they are primarily responsible for relaying this information. This is consistent with previous studies indicating parents experience anxiety and uncertainty when told of an abnormal screen result, and that both improved provider knowledge and methods of communication are needed [12,13]. Families deserve to be empowered with readily available, accurate, and appropriate information and resources to help them navigate the implications of an abnormal NBS result in their infant. We identified the need for development and dissemination of improved educational resources regarding inherited metabolic disease and follow-up for NBS staff and PCPs, in addition to families. 

Examples of web-available resources include the STAR-G Project, and NBS Connect. STAR-G, maintained by the Western States Genetic Services Collaborative, provides general information on NBS, an overview of genetics, and disease-specific fact sheets for both providers and families [9]. Given the wealth of information on this website, it is concerning that only three states reported utilizing STAR-G resources in short-term follow-up. The purpose of NBS Connect, launched by Emory University in 2011, is to collect and analyze primary data on diagnosis, treatment, symptoms, outcomes, and barriers to care in newborn screening disorders [14]. It also provides professional support resources for patients and families. The registry offers unique features such as educational materials, modified recipes, interactive health tracking tools, and opportunities to connect with experts. Currently disorder-specific profiles are available to patients with PKU and Maple Syrup Urine Disease (MSUD) and Tyrosinemia [14]. NBS Connect plans to expand their program to provide a common platform for other disorders recommended by the ACMG for inclusion in the newborn screening panel [5]. In addition to such efforts, other uses of technology, such as creation of apps regarding disorders identified on NBS for PCPs and families should be explored. Previous studies have shown smartphone apps to be effective in assisting patients with disease management [15,16].

Our survey results must be considered preliminary, given the small number of states/territories involved in the SERC. However, we feel our findings are representative of this region as the response rate was 82% and at least one individual from all 10 states/territories completed the survey. Another limitation of our findings is that only the perceptions of NBS staff were obtained with respect to adequacy of knowledge and educational needs/resources. While the perceptions of these individuals may vary by years of experience and familiarity with the NBS process in their state/territory, 11 out of the 14 respondents had five-or-more years of professional involvement with NBS. Expansion of the survey administration to the remaining NBS short-term coordinators in the U.S is planned to provide a more comprehensive look at variation in NBS across the country. Additional studies should be conducted to query PCPs directly regarding their knowledge base and educational needs/resources, and to determine the effectiveness of various means of dissemination of this information. 

The need for readily available, accurate, and comprehensive information and resources for both providers and families will undoubtedly grow as the number of conditions being added to NBS continues to increase. Given the ultimate possibility of NBS whole genome/exome sequencing [17], states/territories and regions need to band together to create these resources in a consistent and anticipatory fashion, and with greater efficiency. Insofar as there are already concerns regarding the adequacy and training of NBS staff and the availability of specialists who are knowledgeable in the care of children with metabolic conditions, efforts should also be directed at increasing the workforce capacity of states/territories to address these anticipated changes.
